# A Quantitative Particle Identification (QPID) spectral autoradiography system

**DOI:** 10.1038/s44172-025-00426-1

**Published:** 2025-05-15

**Authors:** Stephen S. Adler, Noriko Sato, Kwamena E. Baidoo, Frank I. Lin, Woonghee Lee, Colleen P. Olkowski, Freddy E. Escorcia, Peter L. Choyke

**Affiliations:** 1 Clinical Research Directorate, Frederick National Laboratory for Cancer Research, Frederick, MD USA; 2 Molecular Imaging Branch, National Cancer Institute, Bethesda, MD USA

**Keywords:** Imaging techniques, Radiotherapy

## Abstract

Autoradiography is used to study the distribution and binding of radioisotope tagged ligands in tissue at microscale among other applications. The technology has evolved since its inception when it used analogue film exposure techniques with the introduction of digital imaging systems sensitive to ionizing radiation. We report on the development of our Quantitative Particle Identification spectral autoradiography system (QPID), which is based on the Timepix3 sensor and a gamma detecting scintillation crystal. This autoradiography system leverages the ionizing radiation detection features of the Timepix3 to measure the energy deposition from charged particles from radioisotopes with a time resolution of 7.7 ns full width at half max (FWHM), generating spectral or activity autoradiography images. The QPID includes a scintillation crystal used to record gamma emissions coincident with the Timepix3 ionization events with a time resolution of 24.2 ns FWHM. The QPID can separate tracks between α and ß particles, select specific ranges of deposited energies or select on the presence of coincident gamma emissions within a selected energy range when generating images. The QPID has a 10% linearity response up to 700 Bq for 223Ra and 2.5 kBq for 18 F radioisotopes. Using α and ß+ particle identification filters, separate images of ^223^RaCl_2_ and Na^18^F activity distributions were generated from a bone sample infused with the two radioligands together. This unique capability can open the door to the study of targeted radiotherapies which use theranostic α and ß+ imaging agents by measuring their relative pharmacokinetic properties.

## Abstract (web summary)

Stephen Adler and colleagues report on a spectral digital autoradiography device based on a Timepix3 sensor and an external CeBr3 crystal. It has the capability of using gamma and charge particle coincidences to generate radioisotope specific images

## Introduction

From its inception, autoradiography has relied on a time-based exposure of radiosensitive material to radioactive samples. Becquerel in 1896 is credited with recognizing the potential of autoradiography when he noticed that a photographic plate was exposed by a sample of uranium^1^. Today, the most common autoradiography systems work by exposing the surface of a radiosensitive material, in the form of a plate or film, to a radioactive sample. The exposure levels result in an image of the distribution of radioactivity on the surface of the sample.

Autoradiography has since evolved to include new digital methods of detecting ionizing emissions from radioisotopes on the sample’s surface^2–4^. Of these methods, two of the more recent developments are relevant to our work. The first method detects alpha emissions with a ZnS(Ag) scintillator as the radiosensitive film in conjunction with a complementary metal-oxide-semiconductor (CMOS) image sensor, commonly used in digital cameras^4,5^. The image sensor records the scintillation light caused by the alpha particles penetrating the scintillator film after it has been amplified by a microchannel plate (MCP) detector. These CMOS-based systems have exposure times on the order of 10 ms and typically record a single to a few charge particle emissions per frame. The ionizing-radiation quantum imaging detector (iQID) line of products use this type of technology (QScint, Tuscon, AZ). The second method uses a micropattern gaseous detector called parallel ionization multiplier (PIM)^6,7^. The sample is placed on the inside top surface of a gaseous chamber in which a high-voltage electric field is present. The α and β particles emitted from the sample ionize the gas. The ionization creates free electrons and positive ion pairs, that drift under the influence of the applied electric field, creating a current signal on individual pixels of the pixelated reading floor located on the opposite side of the chamber, generating the image. These systems have detection times on the order of µs. Because of the short detection time, typically only one charge particle emission is detected at a time. This technology can be found in the BeaQuant product line (AI4R, Nantes, France).

The Conseil Européen pour la Recherche Nucléaire (CERN) knowledge transfer (KT) group^8^, through its Medipix collaboration, has developed a sensor designed around a pixelated solid-state detector and a high-speed readout application-specific integrated circuit (ASIC) using time over threshold discriminator detection techniques^9^. Of the various models developed by this collaboration, the Timepix3 model^10^, a 256 × 256 pixelated, 300 µm thick Si solid detector, with a 55 µm pitch, represents a potential paradigm shift for autoradiography (Supplementary Fig. S1a, b). This model can read out each pixel independently providing time and energy information for each pixel ionized by the passage of a charged particle through the sensor’s ionization volume. When active, a stream of pixel data is acquired by the Timepix3 ASIC including which pixel was ionized, the energy deposition and the detection time with a least bit sampling resolution of 1.5625 ns. Using time aggregation algorithms, one can reconstruct ionization tracks by collecting near adjacent pixels that have the same detection time within a time window as short as 50 ns. From the reconstructed track information, we can measure the energy deposited by the charged particle and the time when the particle track was detected.

What makes Timepix3 stand apart from the current generation of digital autoradiography systems, is the ability to form coincidences between the particle tracks recorded by the Timepix3 sensor and gamma emissions recorded by the separate scintillation crystal with a coincidence time window as small as 60 ns. This provides a method to identify nuclear decay pathways of the radioisotope by using an energy threshold of the coincident gamma emission. Another feature is the ability to distinguish between ß^+^ and ß^−^ emissions by selecting ß^+^ identified ionization tracks which are coincident with 511 keV gamma emissions following annihilation.

The particle track recording capabilities of the Timepix3 allows for the reconstruction of autoradiography images in a variety of ways. First, we can reconstruct an activity image in units of event rate per area or a spectrographic image in units of deposited energy rate per area. For either case, we can reconstruct the image from the full particle track, called an event map image, or from the centroid of the track called a centroid image. These reconstruction methods allow us to generate images which can measure two distinct properties of the radiation. Activity images which are linear with the activity in the sample can be scaled to units of Bq per area with an appropriate calibration factor. Similarly, spectroscopic images which are linear to the deposited energy can be scaled to units of keV per second per area with an appropriate calibration factor. The former can be used when measuring uptake of a radioactive compound relative to the amount of injected activity for biodistribution and pharmacokinetic studies. The latter can be used to estimate the absorbed dose to tissue needed in microdosimetry studies. Event map versus centroid images affect overall image quality and each type of image is appropriate for different applications which will be discussed later.

To demonstrate the imaging capabilities of QPID autoradiography, we performed two studies with this device. First, we prepared ^89^Zr-oxine-labeled T cells, which were injected intravenously in mice. We then harvested the spleen and measured the labeled cell concentration distribution throughout the organ. Imaging in vivo the microdistribution of cells in organ systems allows for investigators to draw inferences about the microenvironment of tissue from anatomical imaging. The second study involved measuring two compounds that have tropism for bone, ^223^RaCl_2_ and Na^18^F, by co-injecting them in mice, harvesting bone, then generating separate images of ^223^Ra^2+^ and ^18^F^−^ in tissue and measuring their relative uptake, or bioequivalence. We show that QPID can distinguish radiation emission types in situ and demonstrates proof-of-concept microdosimetry of tissues, allowing the possibility to correlate micro- to macro-scale dosimetry. This is needed for personalized radiopharmaceutical therapy which is currently an unmet need for clinical translation.

## Results

### QPID description and its performance

**Fig. 1:**
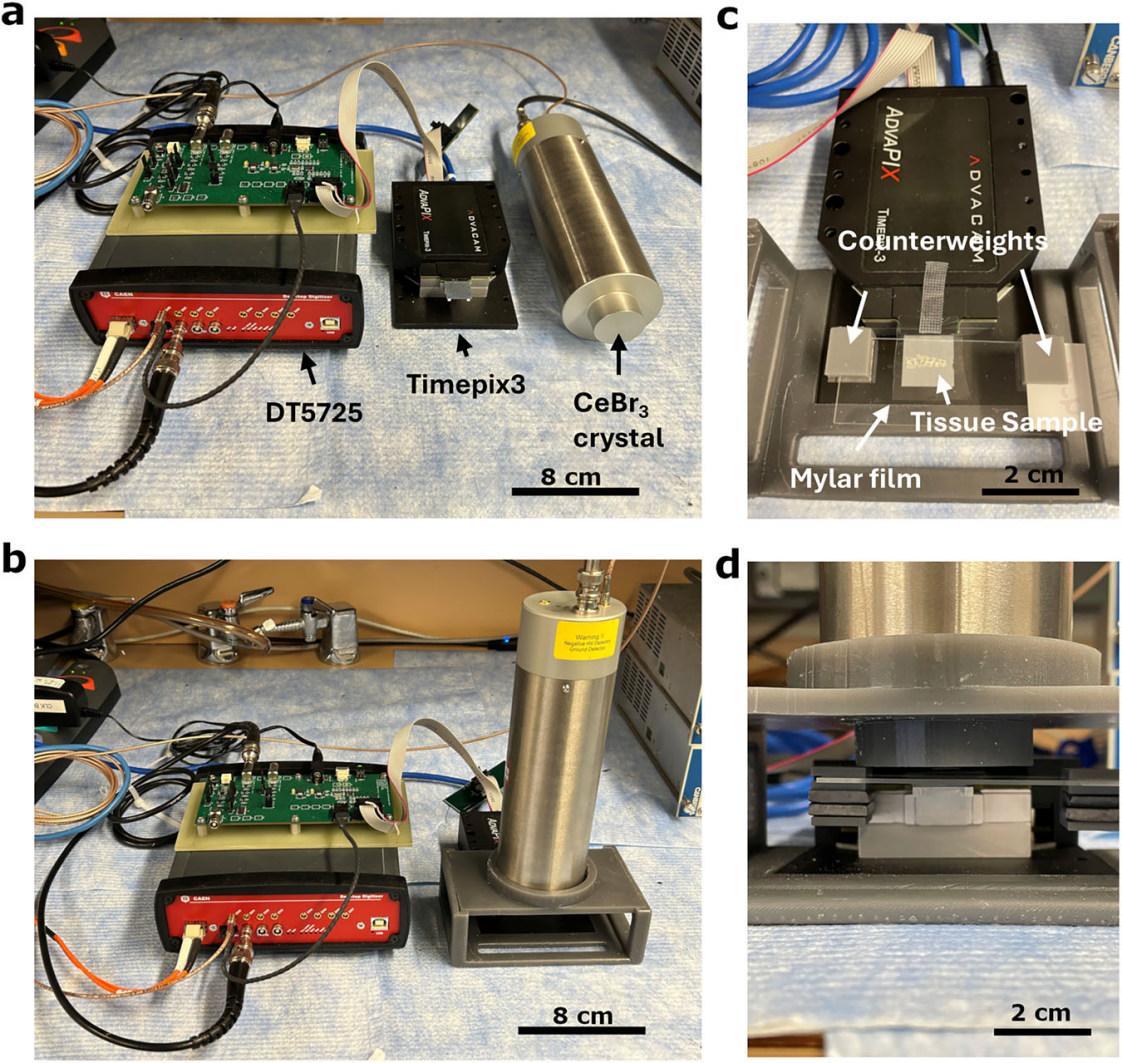
Photos of the QPID setup. **a** The equipment used to collect the data including the Advacam Timepix3, the Berkeley Nucleonics CeBr_3_ crystal detector, the CAEN DT5725 250 MSamples/s digitizer and the external clock source providing synchronized 50 MHz and 40 MHz external clocks to the digitizer and Timepix3 unit, respectively. **b** The QPID in data taking configuration with the CeBr3 detector placed over the source. **c** Spinal microtome sample placed over the Timepix3 sensor with a layer of mylar between the sample and sensor to protect from contamination. **d** A closeup of the CeBr_3_ detector module placed over the sample which is resting on the surface of the Timepix3 sensor.

**Fig. 2:**
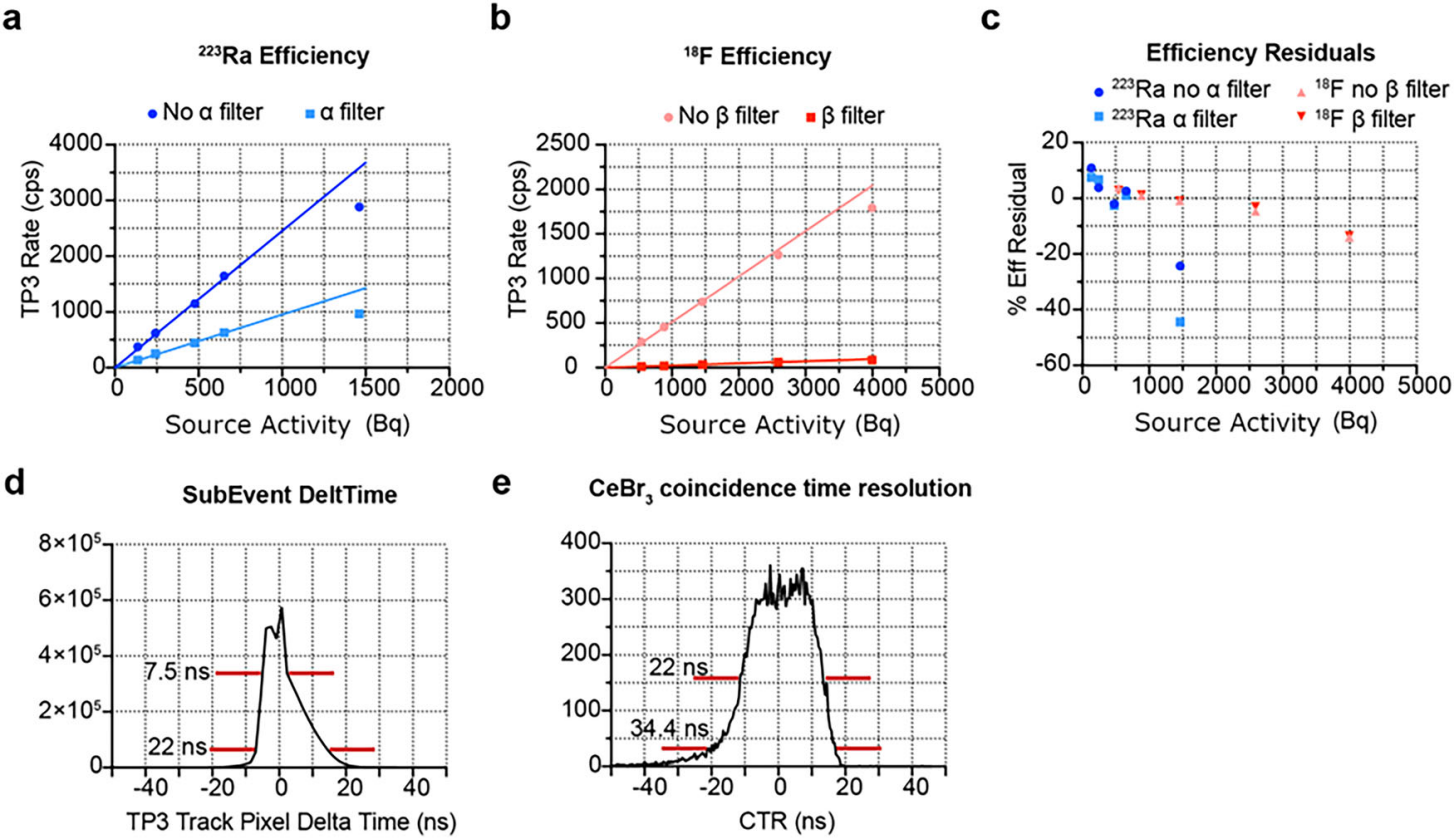
QPID performance plots. **a** Linearity efficiency plots with and without PID filtering for ^223^Ra. **b** Linearity efficiency plots with and without PID filtering for ^18^F. **c** Plot of the % residual of the ^223^Ra and ^18^F efficiency linearity fits. **d** Histogram of the time difference between pixels forming a track. **e** Coincidence time resolution plot of positron tracks and the 511 keV gamma detected by the CeBr_3_ crystal detector.

The QPID includes the Advacam Timepix3 sensor (Prague, Czech Republic), a Berkeley Nucleonics CeBr_3_ detector (Berkeley CA, USA), a CAEN DT5725 digitizer (Viareggio, Italy) and an in-house designed external clock source used to provide a common clock to the Timepix3 and digitizer modules (Fig. 1). Performance measurements were made of the QPID. These include the linearity response of the Timepix3, the ionization track aggregation time resolution and the gamma-charged particle coincidence time resolution (Fig. 2).

The linearity response of the Timepix3 was measured for ^223^Ra and ^18^F up to 700 Bq and 2.5 kBq, respectively (Fig. 2a, b). The detector response shows 10 % linearity up to 600 Bq and 2500 Bq for ^223^Ra and ^18^F, respectively (Fig. 2c). The limiting factor is the readout bandwidth of the Timepix3 device. The discrepancy between the linearity ranges of 600 Bq and 2500 Bq between ^223^Ra and ^18^F is due to the complex decay chain of ^223^Ra (Supplementary Fig. S4a) which emits 4 α and 2 β^−^ particles for each decay of ^223^Ra compared to a single β^+^ for ^18^F.

The slope of the non-PID filtered linearity plots for ^223^Ra and ^18^F are 2.49 ± 0.04 and 0.476 ± 0.005 respectively which measures the detection efficiency for these two radioisotopes. The measured efficiency for ^18^F, a single β^+^ emitting radioisotope can be mostly accounted for by the 2π solid angle acceptance of .5 of the Timepix3 sensor. As for ^223^Ra, with its 6 emissions and efficiency of 2.49 ± 0.04 is close to a 2π solid angle acceptance of 3. One should remember that the surface of the Timepix3 sensor is protected by a layer of Mylar which absorbs a fraction of the alphas. To best understand these efficiencies, a full Monte Carlo simulation analysis is required which will be the focus of future work.

When forming coincidences between the charged particle track recorded by the Timepix3 and the gamma emissions detected by the CeBr_3_ crystal, several factors come into play. First is the ability of the software used to reconstruct ionization tracks from individual pixels using a time aggregation algorithm. The single-track aggregation has a time resolution full width at half maximum (FWHM) of 7.7 ns and full width tenth maximum (FWTM) of 22 ns (Fig. 2d). Because the FWTM is 22 ns, the single-track aggregation time window is set at 40 ns in the track reconstruction algorithm. The FWHM of 7.7 ns, FWTM of 22 ns and the asymmetric shape of the distribution is a result of the various time measurement uncertainties including the sampling time of 1.5 ns, and the propagation of the ionization signal through the Si wafer. This then convolves with the coincidence time resolution (CTR) when forming coincidences with the gamma emissions detected by the CeBr_3_ crystal. This was measured to have a FWHM of 24.2 ns and a FWTM of 34.4 ns (Fig. 2e). Because FWTM was measured to be 34.4 ns, the coincidence time window is set at 60 ns in the reconstruction software. The CTR distribution shows a flat top of about 15 ns which indicates some phenomena related to the ionization of the Timepix3 sensor, the readout electronics or other cause which will be investigated in the future.

These performance results demonstrate that the QPID can handle activity rates well above rates typically found in tissue samples prepared from organs harvested in radioligand biodistribution studies which are in the 10–100 Bq range.

### QPID image generation and particle identification validation

**Fig. 3:**
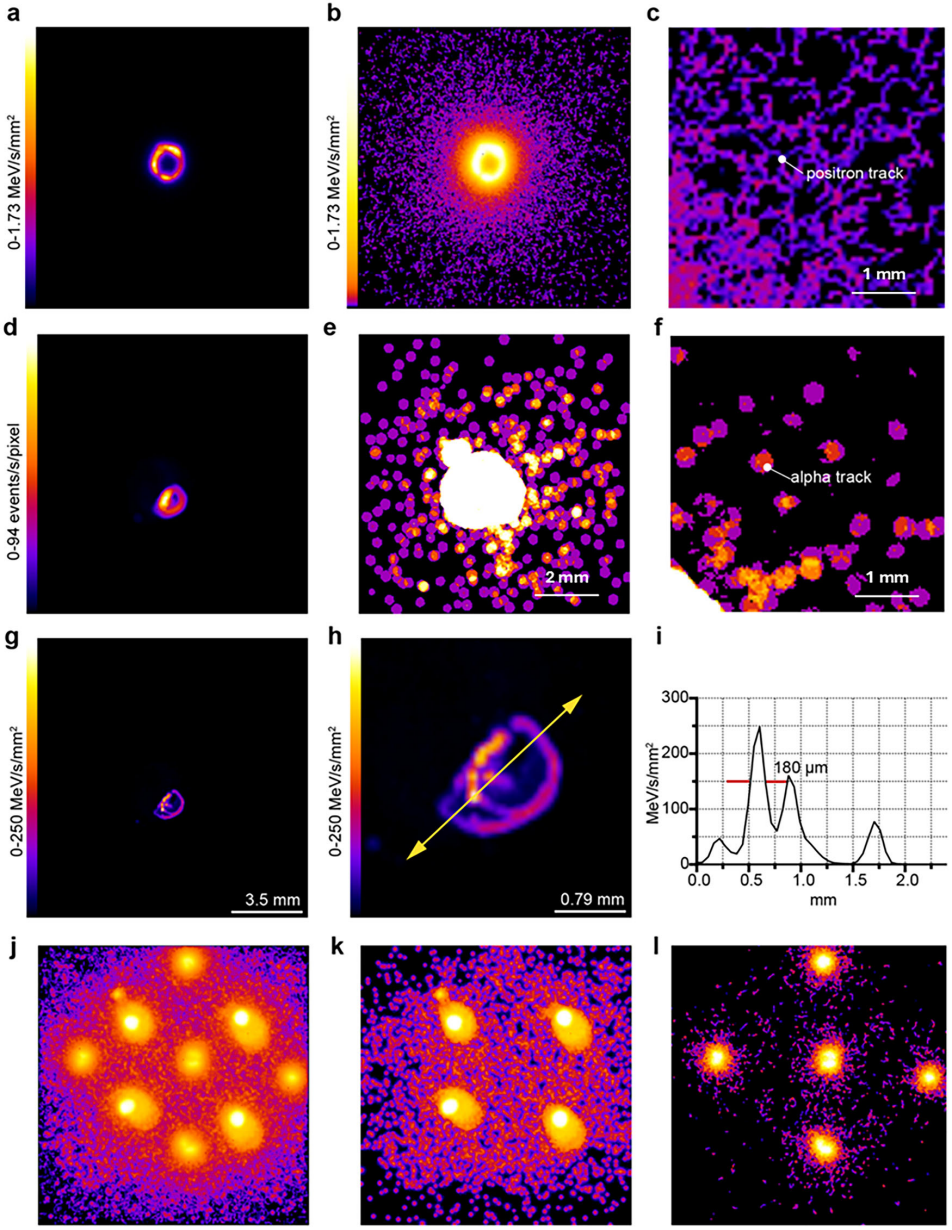
QPID Imaging Samples. **a** A spectroscopic event map image of 1 µL drops of ^18^F. **b**, **c** Log_10_-scaled images of **a** showing the positron tracks from ^18^F decays. **d** An activity rate event map image of 1 µL drops of ^227^Th with the alpha PID event filter applied. **e**, **f** Log_10-_scaled images showing the alpha emission particle tracks. **g** A spectroscopic centroid version of the image (**d**). **h** A magnified version of **g** showing the detail of the ^227^Th residue. **i** A profile through the residue indicated by the yellow arrow in **h**. **j** Activity rate event map image of a mixed set of ^18^F and ^223^Ra 1 µL drops with no PID filters applied. **k**, **l** The same images but with the alpha and positron PID filters applied respectively.

1 µL drops of ^18^F and ^227^Th containing approximately 1 kBq and 100 Bq of activity, respectively, were imaged with the QPID (Fig. 3a–i). Event map activity and spectroscopic images were reconstructed in units of events/s/pixel and MeV/s/mm^2^ respectively to illustrate the versatility of the Timepix3 sensor as a solid-state detector to generate both activity and spectrographic images. Log_10_-scaled versions of the event map images show the particle tracks made by α and particles ß (Fig. 3b, c, e, f). Centroid spectrographic images of ^227^Th illustrates the limits of spatial resolution for α particles (Fig. 3g, h). A profile through the ^227^Th image resulted in the Timepix3 sensor resolving structures with 180 µm FWHM (Fig. 3i).

The distinction between an event map and a centroid image is best seen with the ^227^Th example. The event map image has a very poor resolution because the ionization track of each α particle is circular in shape and in general has a diameter over 400 µm (Fig. 3e, f). But the energy-weighted centroid of the track reflects more accurately the actual position of the α particle and generates a much more detailed image of the source distribution (Fig. 3g, h). The high resolution of the centroid image visualizes the way the 1 µL drop of the ^227^Th solution evaporated and formed the ^227^ Th residue on the microscope slide. Doing the same comparison between centroid and event map image generation for beta particles does not show great improvement since the ionization track is linear with scattering kinks (Fig. 3c). The resolution of beta images is dominated by the beta track range which scales with the energy of the beta emission. Further studies to better determine the imaging resolution of the Timepix3 used in the QPID system are planned.

To test the particle identification features of the QPID, separate 1 µL drops of ^223^RaCl_2_ and Na^18^F were imaged together in a lattice formation. Event map activity images were reconstructed using three different filters, 1) No filter, 2) α filter, and 3) particle track+γ coincidence filter (Fig. 3j–l). The images are displayed at Log_10_-scale to visualize the track of the α and β particles. The QPID was successful in separating the α and β^+^ particle components within the list mode data using these particle identification (PID) filters (Fig. 3k, l).

### ^89^Zr-oxine-labeled T cell study

**Fig. 4:**
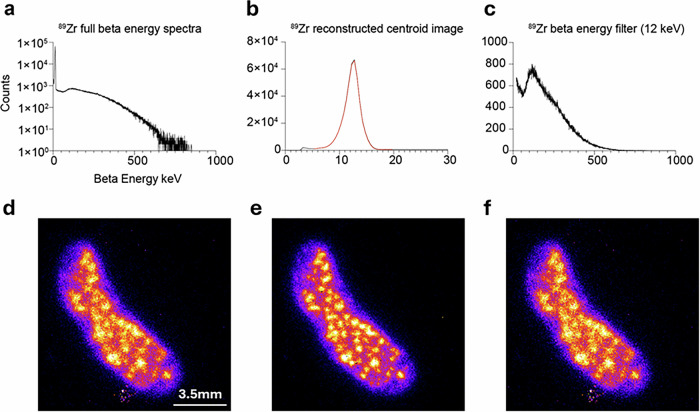
^89^Zr-oxine-labeled T cell images. **a** Plot of the full beta energy spectra measured by the Timepix3 sensor in Log_10_-scale. **d** The reconstructed spectroscopic centroid image with no beta energy filter applied. **b** Plot of the beta energy filter (red) selecting the 12.5 keV beta emissions. **e** Reconstructed image using beta energy filter. **c** Plot of the beta energy spectra for the events not selected by the beta energy filter. **f** Reconstructed image using the beta spectra in **c**. Note there is a difference in the x and y axis scales between (**a**), (**b**) and **c** to better show the components of the energy spectrum used to reconstruct their correspoinding image.

QPID data was acquired from a 10-µm- and 30-µm-thick frozen sections of the spleens harvested from mice which were intravenously infused with ^89^Zr-oxine labeled naïve T cells (Fig. 4). To demonstrate the event filtering mechanism and its effects on image resolution, centroid deposited energy images were reconstructed, along with β emission spectra for the 10 µm spleen sample using different event filtering options. These options are 1) no event filters (Fig. 4a, d), 2) only the 12 keV Auger β tracks using an energy window between 5 to 20 keV (Fig. 4b, e, and 3) Non 12 keV Auger β tracks using an energy filter 20 keV and above (Fig. 4c, f). These set of images demonstrate how one can affect the image quality by applying an energy threshold to the emitted betas from ^89^Zr decay. Because of the nature of the beta emissions from ^89^Zr decay, both β^−^ and β^+^ emissions are present, with an abundance of low energy Auger K β^−^ emissions at 12.7 keV and greater energy β^+^ emissions with an average energy of 395.5 keV and an endpoint maximum energy of 902 keV (Fig. 4a–c). Applying an energy event filter which selects the 12.7 keV tracks (Fig. 4b) filters out the longer ionization tracks producing a sharper image (Fig. 4e). Reconstructing the image using the decays with ionization track energies greater than 20 keV one can see the degradation in image resolution caused by the longer positron ionization tracks (Fig. 4f).

**Fig. 5:**
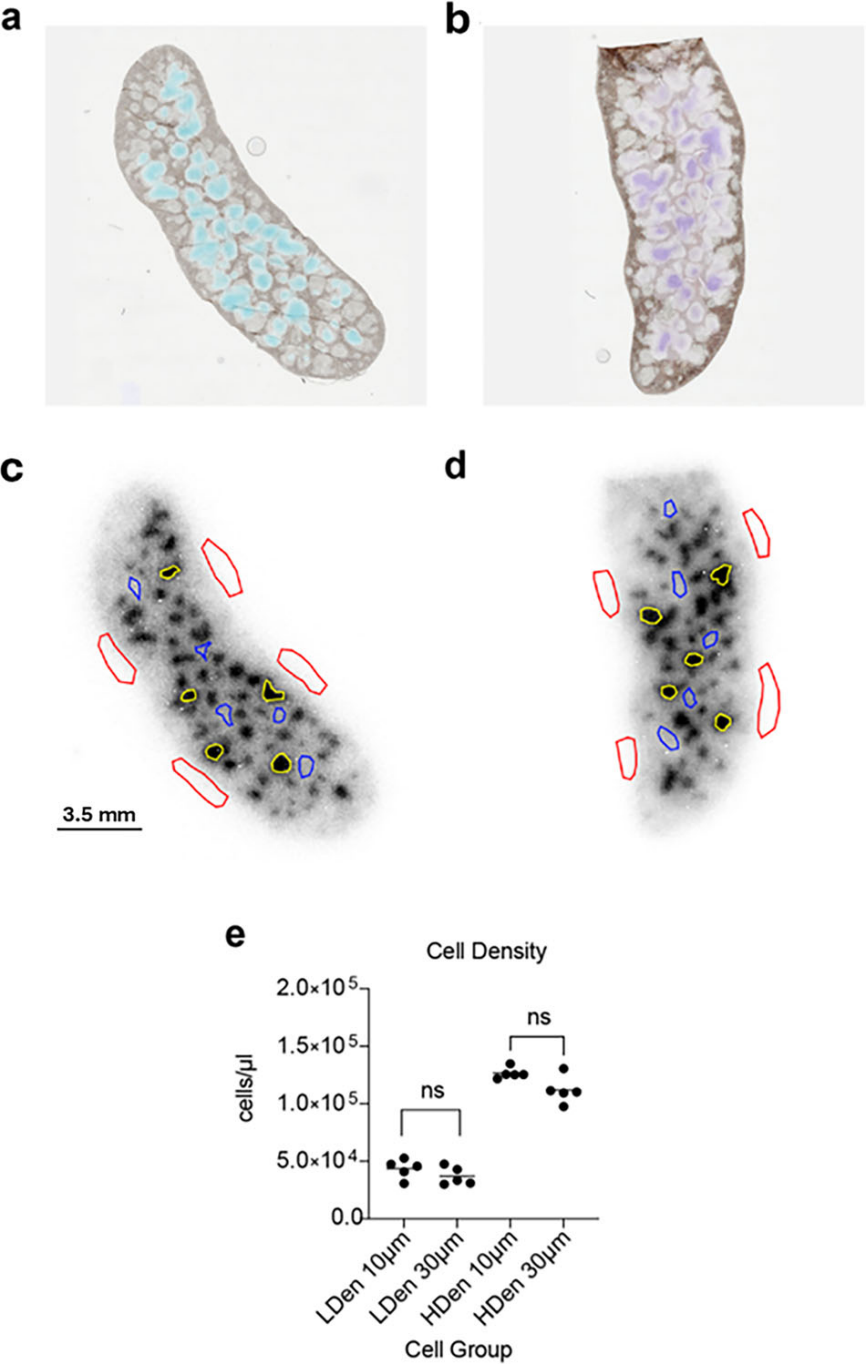
^89^Zr-oxine-labeled T cell density measurement results. **a**, **b** Co-registered QPID and brightfield microscopy images of 10 µm and 30 µm thick spleen tissue samples, respectively. The co-registered images show how the T cells concentrate in the T cell zone of the white pulp. **c**, **d** Location of the regions of interest (ROI) used to measure the background activity (red), high-density activity (yellow), and low-density activity (blue). **e** Results of the activity measurements converted to cell concentration.

QPID images of both the 10-µm- and 30-µm-thick sections were co-registered with their corresponding brightfield images (Fig. 5a, b) showing how the T cells accumulate in the T cell zone of the white pulp. Regions of Interest (ROIs) were drawn on the QPID images to measure cell density levels within the spleen (Fig. 5c, d). Their density in the T cell zone were (127 ± 5) ×10^3^ cells/µl and (112 ± 12) ×10^3^ cells/µl respectively with no significant difference measured between the two. The cell concentration in the regions between the areas of high concentration were (44 ± 8) ×10^3^ cells/µl and (37 ± 8) ×10^3^ cells/µl respectively showing no significant difference between the two samples (Fig. 5e). These results demonstrate the quantitative capability of the QPID with the ability to measure the distribution of ^89^Zr activity within the spleen tissue. The images were reconstructed as activity images allowing conversion of the image in events/s/pixel into Bq/pixel. Using the cell labeling specific activity and sample thickness, we can further convert the images to number of cells per µL. Both mice were infused with the same number and concentration of T cells, resulting in a similar low and high activity density (e.g. cell density) measurements in the two samples. This attests to the reproducibility of the overall procedure which includes the linear response of the Timepix3 sensor. This type of quantitative information can be used as input to immune cell physiology models.

### ^223^RaCl_2_ and Na^18^F bioequivalence study

**Fig. 6:**
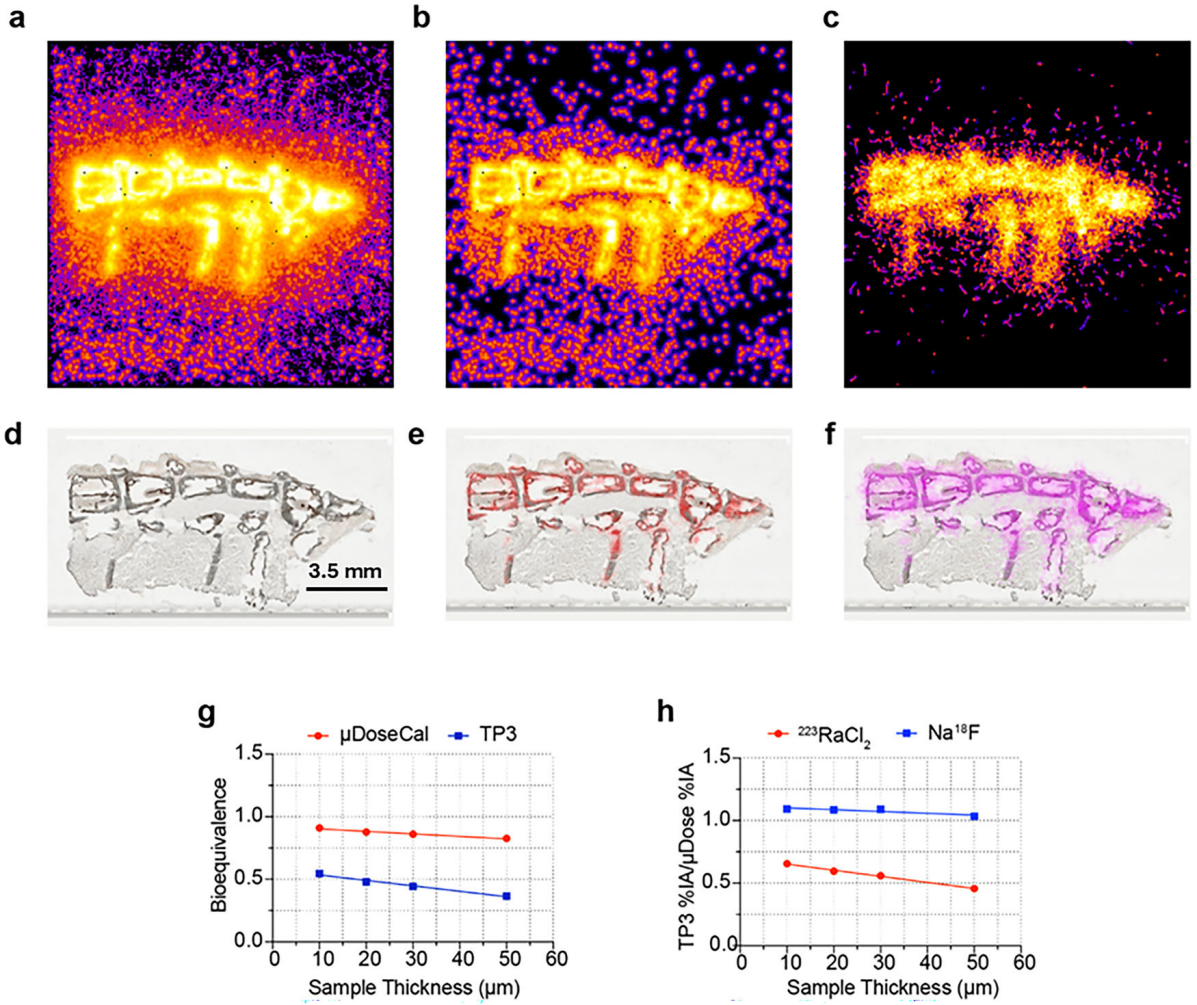
Na^18^F and ^223^RaCl_2_ bioequivalence results. Shown here are representative sets of images from a single list mode QPID acquisition of spine microtome sample containing both ^223^RaCl_2_ and Na^18^F. **a** The reconstructed image without applying any PID filters. **b**, **c** Image reconstructions from the same list-mode data set but with alpha and positron PID filters applied, respectively. The images are displayed in Log_10_-scale to better visualize the alpha and beta ionization tracks. **d** brightfield microscope image of the spine microtome sample. **e**, **f** The alpha and positron image set co-registered with the brightfield image (**d**), respectively. These images are displayed in linear scale. Note, there are a few dead pixels which account for the black speckles which can be seen most notably in **a**. **g**, **h** Plots of the dependence of the bioequivalence and dose comparisons between Na^18^F and ^223^RaCl_2_ and the QPID and microdose calibrator as a function of sample thickness, respectively.

Spine samples were collected from mice co-injected with Na^18^F (7.4 MBq) and ^223^RaCl_2_ (111 kBq). The bioequivalence of the two theranostic compounds^11–13^, defined as the ratio of ^223^Ra^2+^ percent injected activity (%IA) to ^18^F^−^ %IA, was measured as a function of sample thickness at 10 µm, 20 µm, 30 µm and 50 µm with the QPID and a microdose calibrator^14^. The α and particle track + γ coincidence filters were used to separate the QPID data into ^223^Ra^2+^ and ^18^F^−^ images used in the bioequivalence measurements (Fig. 6a–c). Co-registration of the ^223^Ra^2+^ and ^18^F^−^ QPID images with brightfield microscopy images indicates both radio-ions accumulate into the bone (Fig. 6e, f). The bioequivalence as measured by the QPID is less than that as measured by the micro-dose calibrator (Fig. 6g). The negative slope of the bio-equivalence function shows a strong dependance on slice thickness. Separate measurements of the activity ratio of the QPID to the microdose calibrator indicate that the lower bioequivalence is due to fewer alpha emissions than expected (Fig. 6h). On average, the QPID measures a factor of 7.6% ±2.8% higher Na^18^F activity than the microdose calibrator while for ^223^RaCl_2_ the microdose calibrator measures on average 79% ±8.3% higher activity compared to the QPID measurement. Doing a linear regression analysis on the ^223^RaCl_2_ data set (Fig. 6h) results in a slope of −0.0049 ± 0.0002 µm^−1^ and a y-intercept of 0.701 ± 0.007 (R^2^ 0.9958). Because ^18^F is a reasonable imaging surrogate for therapeutic ^223^Ra distribution, these data show the ability of the QPID to measure the effects of bone attenuation on alpha emissions from ^223^Ra decays, leveraging the ^18^F data collected at the same time. To note, we are assuming that most of the alpha absorption or attenuation is due to bone tissue since the ^223^Ra is absorbed by the bone. Future studies will measure the absorption of alphas in soft tissue.

## Discussion

Here, we report on a new class of autoradiography system, QPID, which extends beyond the capabilities of current technologies. QPID is a digital spectral autoradiography system coupled with an external gamma detection crystal that allows us to harness the data from the imaged sample in unique ways. At a basic level, the linear response of the QPID, which is within the range typical for biodistributions of up to 100 Bq and 500 Bq for α and β emitting radioisotopes, respectively, makes it simple to make quantitative activity and spectroscopic measurements. We demonstrate that we can use the spectroscopic data to enhance image quality with the T cell imaging study by selecting only the ^89^Zr 12.5 keV Auger decays to reconstruct the image. With its linear response, we were able to then measure pertinent T cell density distribution data within a spleen. This can be extended to other studies of microdistribution of radiolabeled cells or radioisotopes in other organs. Using spectroscopic and topographic data, we can perform particle identification, distinguishing tracks being made by either α or β particles. We can further refine the particle identification capabilities to identify β^+^ with a high degree of accuracy by incorporating the coincident data from the gamma detection crystal. Finally, we generated separate radioisotope images from a sample containing both an α- and β^+^-emitting radioisotopes, giving us a powerful tool to study the interaction of the two within a tissue sample. For dosimetry, when we combine the spectroscopic and particle identification capabilities of the QPID we can now identify the type of ionizing radiation contributing to the absorbed dose of the tissue being studied enabling assessment of multiple radioisotopes concurrently. Such work can help bridge the gap between microdosimetry and macrodosimetry and improve our ability to personalize radiopharmaceutical therapy for patients with cancer.

The work presented here is only possible because Timepix3 sensor has the unique ability to independently readout the time and ionization energy deposition from a particle track within each individual pixel with a sampling time of approximately 1.5 ns. We reconstructed particle tracks with a time resolution of 22 ns FWTM and use them to form coincidences with gamma emissions with a time resolution of 34.4 ns FWTM with a 60 ns coincidence time window. The 60 ns coincidence window sets the scale for the rate at which one can operate the QPID. This is best done by doing a simple coincidence signal to noise estimate where the noise is made up of random coincident events. Typical rates for the ^223^Ra^2+^ and ^18^F^−^ dual isotope study were on the order of 100 events/s recorded by both the Timepix3 and the CeBr_3_ crystal with a coincidence rate of about 10 events/s. The estimated randoms count rate using a 60 ns coincidence window is 0.0006 events/s resulting in a signal to noise ratio of 17 × 10^3^. What is more important is this type of single decay event selection with gamma coincidence tagging cannot be done with conventional film-based analogue autoradiography systems. For the newer CMOS based ZnS(Ag) autoradiographs used in alpha cameras like the iQID^4^, detection of the coincident gamma ray is difficult because the exposure times are on the order of tens of milliseconds. To understand this in context, if one were to use a 10 ms coincident window with the QPID, the resulting signal to noise ratio is 0.1 compared to 17 × 10^3^ for a 60 ns coincidence time window. The iQID coincidence data would be overwhelmed by randoms background at a ratio of 10 random background events to 1 true coincident signal event. While gamma emission coincident tagging can theoretically be done with PIM autoradiography systems, it has not yet been demonstrated.

In addition to exploiting the timing characteristics of the Timepix3 to form coincidences with gamma emissions, the Timepix3 also records deposited energy directly allowing us to reconstruct spectrographic autoradiographs. This can be used to measure absorbed dose in tissue directly at the microscale of the autoradiograph. For β-emitting isotopes, generating event map spectroscopic images results in a dose map distribution, which includes the full track of the electron. To achieve more accurate absorbed dose estimates, some correction factors are required and include accounting for different linear energy transfer (LET) and range of the emitted electron between the silicon wafer where the ionization is measured by the Timepix3 and the tissue where the radioisotope is present. One great advantage of the QPID is that we can do this conversion on an individual particle track basis producing a more accurate radiation dose distribution as opposed to relying on kernel convolution techniques applied to the final image. A similar technique can be used for α-emitting radioisotopes. While the effects of attenuation in tissue and the more complex energy calibration make doing alpha dosimetry more challenging, we demonstrate that it is feasible. Optimizing these microdosimetry methods are planned for future research.

Being able to use the full track length of the decay emission particle to model dose deposition and distribution give us an advantage in dosimetry estimates when compared with the iQID (ZnS(Ag)) and PIM (gas chamber) autoradiography systems. The iQID system uses a convolution kernel method to convert its activity image to a dose image^15^. PIM-based autoradiography systems do have spectral imaging capability for alpha radioisotopes which can be used for dosimetry estimates^7^ but relies on the LET of the track as it enters the gas chamber as its input to its dose model.

With respect to particle identification features, both ZnS(Ag)-based (iQID) and PIM-based autoradiography systems do have the capability of distinguishing α from β particles. However, they are not able to perform dual isotope studies like the ^223^RaCl_2_/Na^18^F study we presented here. Most α emitting radioisotopes decay by both α and β^−^ decay which, in effect, floods the data with β^−^ backgrounds. If you try to image both an α and β^+^ emitting isotope, one will not be able to distinguish the β^+^ emitted from the β^+^ radioisotope from the β^−^ emitted through the decay chain of the α radioisotope. Detecting the coincident 511 keV annihilation gamma is critical to separate the α emitting radioisotope from the β^+^ one and only possible with the QPID. The detection efficiency for β^+^ using the coincident method is only 2.3%, measured from the ^18^F linearity data, but it is still sufficient to be useful. Future designs of the QPID can increase the solid angle coverage of the gamma detection crystal and therefore increase the coincidence detection efficiency.

What makes dual isotope studies so compelling is that measuring the absorbed dose as a %IA ratio of the two compounds in a co-injection study cancels out many biological factors. For example, the bone and tissue mass cancels out in the ratio. If one were to measure the two radioactive ions independently, it would require measuring %IA of gram of the tissue (%IA/g) for each. Although measuring the mass of bone spicules and tissue in a microscopy sample is feasible, it would be very difficult and add uncertainty to the resulting measurement.

Co-injection studies of α and β^+^ emitters is the best example of how the QPID can be leveraged to study the relative bio physiology of two related compounds with different radioisotopes because of how well the QPID can distinguish the two. While β^−^ and β^+^ emitting co-injection studies can be performed, trying to distinguish a β^−^ from a β^+^ will be more difficult and require further study of the analysis technique. This is because the track of β^−^ and β^+^ particles are identical, and one would need to look at β^+^+γ emission while studying β^−^ as background events. This is relevant if trying to assess colocalization of ^177^Lu based cancer treatment agents like ^177^Lu-dotatate with its imaging pair ^68^Ga-dotatate.

Research efforts attempting to multiplex imaging radioisotope, that is simultaneous imaging of different radioisotopes localizing to distinct targets have been explored. For example, Pratt et al. used two different β^+^ emitting isotopes imaged at the same time, resulting in two distinct image sets for each radioisotope^16^. This macroscopic scale imaging, where dual and triple coincidence tagging is used to distinguish between the two radioisotopes requiring advanced positron emission tomography (PET) scanners capable of triple coincidence tagging. These studies are different to what we are reporting here since the two different β^+^ radioisotopes do not form a theranostic pair used in radiophamaceutical therapy. The QPID images at the microscopic scale can image theranostic radioisotope pairs simultaneously.

Another use case would be to study the daughters in the decay chain of an α emitter. For example, ^225^Ac decay daughters ^221^Fr and ^213^Bi can be selected by tagging the 218 keV and 440 keV coincident gamma emissions. This can lead to studies of how decay daughters affect micro-dosimetry models since the major component of the dose is due to alpha decays from the daughters.

The work presented here is preliminary showing the capabilities of the QPID. There is much work to be done before it can be used for dosimetry studies. To start, a more systematic characterization of the Timepix3 energy, timing and spatial resolution are required. We do not have enough data to properly characterize the minimum energy threshold other than the manufacture’s report of 2.2% at 23 keV with a minimum energy threshold of 3 keV, measured with x-rays. This should be measured with α and β particles. These measurements will be difficult, especially for α particles, because they are best performed in the vacuum cell of an alpha spectrometer. To properly use the data from the QPID for in tissue micro-dosimetry measurements, a comprehensive Monte Carlo simulation system needs to be developed. With it, one can extrapolate the energy deposition measurement from tracks made in the Si wafer to the energy deposition in tissue. Some of the issues which need to be address when making these dose estimates to tissue are cross-dose effects, heterogeneity density in tissue and extrapolation from single point dose measurement to full time distribution of the activity within tissue. These are complex topics which will be address in future work. To note, one cannot do full micro-dosimetry with the QPID, but it can be used to help validate dosimetry models within a single timepoint of the pharmacokinetic distribution in tissue.

In sum, QPID extends beyond traditional autoradiography techniques bringing together event rate measurements, dose estimation and unique particle identification capabilities provided by its coincident gamma detection feature. This in turn, opens the door to more sophisticated analysis of autoradiographic samples, including but not limited to the ones highlighted in our study. As such, QPID gets us closer to multi-radionuclide microdosimetry in tissue, which is important to establish dosimetric models across the micro and macro scales—a critical step toward developing accurate personalized dosimetry in patients receiving radiopharmaceutical therapy.

## Materials and methods

### QPID design

The **Q**uantitative **P**article **Id**entification autoradiography system (QPID) is composed of a pixelated Si solid state detector and a CeBr_3_ scintillation crystal both synchronized in time by a common external clock source. The solid-state detector is the Timepix3 module purchased from Advacam (Prague, Czeck Republic) (Fig. 1a). It is made up of a 300 µm thick Si wafer for optimal α particle detection as suggested by Advacam when deciding which model to purchase for this project. The other choices were GaAs, CdTe, and CZT. It sits on top of a 256 × 256 grid of pixel contacts which interface to the Timepix3 readout chip (Supplementary Fig. S1a). The solid-state detector is sensitive to α and β particle ionizations when a bias voltage is applied between it and the Timepix3 readout ASIC. The pixel contacts are spaced 55 µm apart which form an imaging field of view of 1.4 × 1.4 cm^2^. The Timepix3 readout ASIC operates by measuring the time of arrival and time over threshold of the ionization pulse caused near one of the pixel contacts (Supplementary Fig. S1b). Each pixel is readout independently into a list mode data stream. The Timepix3 unit is operated using a software package called pixet provided by Advacam which allows one to configure the operation mode (frame vs pixel readout), the data output files specifications, selecting the clock source and start signal (internal vs external).

The CeBr_3_ crystal detector module uses a Hamamatsu R13089-100 PMT operating at −1.3 kV purchased from Berkeley Nucleonics (San Rafael, CA) (Fig. 1a). The CeBr_3_ PMT detector module is readout with a CAEN (Viareggio, Italy) DT5725 250MS/s 14 bit digitizer (Fig. 1a). The digitizer is operated by a software interface developed in-house. The software sets the digitizer in external clock mode and configures it to generate an external start pulse when the digitizer starts taking data. The digitizer software interface also writes out a list mode data file for each data packet read from the digitizer storing the time and energy of each gamma detected by the CeBr_3_ crystal.

Both the Timepix3 and DT5725 have external clock inputs used to time synchronize the two devices to enable charge track and gamma coincidence detection. The DT5725 requires a 50 MHz external clock while the Timepix3 requires a 40 MHz clock. The Timepix3 has an external start trigger input needed to synchronize the acquisition start time. An external clock board was designed using a 200 MHz clock (Supplementary Fig. S1c). Two of the clock outputs are divided down by a factor of 4 and 5 to generate 50 MHz and 40 MHz clock outputs, respectively. These are fed into the external clock inputs of the DT5725 and Timepix3, respectively. The clock board also receives the external start output signal from the DT5725 and feeds it into the Timepix3 external start input signal (Supplementary Fig. S1d).

### Sample placement

To maximize sensitivity and image quality, it is important to make sure the tissue sample is in contact with the Timepix3 silicon wafer surface. This is done by placing an 18.9 micron thick Melinex S polyester mylar film (Tekra, New Berline, WI) on the Si wafer detector surface to protect it from contamination from the tissue sample. To ensure good contact, two lead counterweight holders were made with a 3D printer, fitted with 1/4” thick lead pieces, and slotted onto the ends of the microscope slide. The microscope slide is then placed with the tissue sample directly resting on top of the mylar film protecting the Timepix3 detector surface (Fig. 1c).

### QPID data acquisition and image reconstruction

The QPID uses two software interfaces to generate data. The pixet software to operate the Timepix3 unit and an in-house written user interface to operate the DT5725 digitizer. An imaging session consists of operating the two software interfaces at the same time, each generating their own list mode files, one containing the data collected from the Timepix3 sensor and the other from the digitizer reading out the CeBr_3_ crystal data.

The algorithm used to generate the autoradiography image starts by parsing the pixet list mode file collecting the ionized pixel index, the time over threshold measurements and time the pixel was ionized. Ionization tracks are assembled by looking for near-adjacent pixels which were ionized within a coincidence window of 40 ns (Fig. 2d). From the assembled list of pixels which make up a particle track, the energy and time of the event are estimated. Advacam supplies a factory calibration file which converts the time over threshold measurement to a deposited energy in keV. Using this calibration file, the deposited energy is calculated by summing the energy of each pixel in the track in keV. The global ionization time of the track is the average of the ionization times of the track pixels. The position of the track is the energy-weighted centroid of the track pixels.

When forming coincidences, the list mode data file generated by the DT5725 digitizer is read in extracting the time and pulse area for each gamma event. The pulse area is converted into an energy using a calibration constant determined from a mixed gamma energy source. A coincidence algorithm finds events in the DT5725 list mode data which are within 60 ns of an ionization track reconstructed from the Timepix3 list mode data file. Those events are tagged as coincident and the time and energy of the DT5725 event packet are stored along with the particle track data. This information is then used by the event selection filter algorithms.

Images can be reconstructed as event map or centroid images, and each scaled in units of event rate per area or in units of deposited energy rate per area. Event map images are made by superimposing the particle tracks of all the events recorded by the Timepix3 sensor. Centroid images are made by taking the energy-weighted centroid of the particle track and superimposing the centroids of all the tracks to generate the image. Images scaled to event rate per unit area are reconstructed by incrementing each track pixel by 1/N for event map images, where N is the number of pixels forming the particle track or by just 1 in the centroid coordinate for centroid images. For images scaled to deposited energy per unit time and area, one replaces the unit increment in the centroid image with the deposited energy and the pixel energy for each pixel forming the ionization track for the event map images.

### Energy determination

As mentioned previously, the Timepix3 has a factory calibration dataset which converts the ionization time over threshold measurement into energy in units of keV. The energy calibration was tested with beta emitting radioisotopes and showed good agreement with expected energy ranges of the beta emissions from ^89^Zr (Fig. 4a–c).

The factory calibration files do not work for alpha ionization tracks. This is due to their high linear energy transfer which saturates the ionization response of the Si substrate resulting in lower deposited energy measurements (Supplementary Fig. S2a). The problem is made worse by the protective mylar film which absorbs about 1.5–2 MeV of the ionization energy as the α particle traverses the film (Supplementary Fig. S2b).

To compensate for these α energy measurement issues, a secondary energy calibration was performed and applied to α identified ionization tracks. The calibration was determined by using an α spectrometer (ORTEC, Oak Ridge, TN) to measuring the energy spectra from ^239^Pu (5.144 MeV), ^241^Am (5.485 MeV), ^244^Cm (5.804 MeV) and the alphas from the ^227^Th decay chain which are ^227^Th (6.038 MeV, 5.756 MeV), ^223^Ra (5.716 MeV), ^219^Rn (6.819 MeV), ^215^Po (7.386 MeV), and ^211^Bi (6.622 MeV). Energy spectra were generated with and without the mylar film placed over the isotope samples. The same isotopes were imaged on the Timepix3 sensor generating energy spectra using the factory provided energy calibration factors. The α particle energy histogram distributions of both the α spectrometer and the Timepix3 sensor allows us to generate an energy transformation function which converts energy measured by the Timepix3 into energy measured by the α spectrometer (Supplementary Fig. S2c). To test the α energy calibration, energy spectra from a ^239^Pu, ^241^Am, and ^244^Cm mixed α source acquired on the alpha spectrometer were compared to the calibrated Timepix3 alpha energy spectra (Supplementary Fig. S2d). This comparison was also done with a ^223^Ra source (Supplementary Fig. S2e) and a ^227^Th source (Supplementary Fig. S2f). One should note that the ^227^Th source used for the calibration validation was purified a few days before the α spectrometer spectra were collected, thus one can see the α emissions from the beginning of the ingrowth of the daughter radioisotopes of the decay chain.

### Particle identification

There are three main algorithms which are used to determine the particle type recorded by the QPID. Energy discrimination, track topology and ionization track plus CeBr_3_ gamma event time coincidence.

β particles typically have much lower energy than α particles when emitted from a radioisotope. β emissions tend to have less than 1 MeV, while α particles tend to have energies much greater than 1 MeV, with 5 MeV being typical. Therefore, using a high energy threshold (HET) of 1 MeV as an event selection filter does a good job of separating β from α tracks.

Because the linear energy transfer (LET) is much larger for α than β particles, the ionization topology is very different. The ionization track recorded by the Timepix3 for α tracks is typically circular (Fig. 3f), while linear for betas (Fig. 3c). Due to scattering, the beta tracks also show a random walk pattern. Therefore, a simple circle versus line or jagged line segment filter is used to identify an ionization track as made by an α or β particle.

To identify β^−^ versus β^+^, a coincident filter is applied requiring a β charged track to be coincident in time with a 511 keV gamma detected by the CeBr_3_ crystal using a lower energy threshold (LET) of 450 keV to select the β^+^ annihilation gamma events.

Using the above selection criteria, the α PID filter requires events to have circular ionization pattern and a LET of 3 MeV. The β^+^ PID filter requires the track to have a HET of 1 MeV and be coincident with a gamma with a LET of 450 keV. There is no need to filter on track topology, so it is not included.

### Microdose calibrator

A microdose calibrator^14^ was used to measure the activity of the radioisotope samples used in the various studies reported in this manuscript. The microdose calibrator is a segmented well counter which has a greater dynamic range and larger sampling well than a typical well or gamma counter used in radionuclide-based pre-clinical studies. The device was designed and built by the NCI/MIB physics laboratory, and its performance has been reported in the literature^14^.

The calibration factors for the microdose calibrator are derived using different methods. For the ^89^Zr-oxine labeled T cell studies, the calibration standard was prepared using a Capintec dose calibrator located in the animal lab where the cell labeling, and mouse studies are performed. Using the Capintec setting for ^89^Zr of 465, a 37 MBq (1 mCi) sample of ^89^Zr was measured in the dose calibrator and diluted into a 1 L volume of water. A 1 mL sample was aliquoted from the 1 L volume and placed in a 1.5 mL centrifuge vial making a 37 kBq (1 µCi) ^89^Zr calibration source used to measure the ^89^Zr microdose calibration factor.

For the dual radioisotope ^223^RaCl_2_ and Na^18^F bio-equivalence study, 37 kBq samples were prepared in 1 µL volumes and placed in a 200 µL vial. The samples were measured by a well type high purity germanium detector (HPGe) (Mirion Technologies, Atlanta GA, USA) calibrated using a NIST traceable mixed gamma source (Eckert & Ziegler, Berlin, Germany). These ^223^Ra and ^18^F samples were then used to measure their respective calibration factors for the microdose calibrator.

To measure the activity mixture of ^223^Ra and ^18^F used in the dual isotope bio-equivalence study with the microdose calibrator, an energy histogram deconvolution technique was employed. The energy spectra measured by the microdose calibrator of a sample containing multiple isotopes is a linear sum of the energy spectra of the radioisotopes plus the background as shown in the Eq. (1).

1 $${S}_{i}={\sum}_{j}{A}_{j}{\varphi }_{{ij}}$$

Where ${S}_{i}$ is the multi-isotope energy spectra histogram for bin number $i$, ${\varphi }_{{ij}}$ is the energy spectra of isotope $j$ for bin $i$ and ${A}_{j}$ is the activity of isotope $j$. ${\varphi }_{{ij}}$ are the basis spectra normalized to unity ($\int \varphi =1$). Then using the MINUIT fitting algorithm^17^, one can find the values of the fit parameters ${A}_{j}$ which minimize the following chi square function.

2 $${\chi }^{2}={\sum}_{i}{\left({S}_{i}-{\sum}_{j}{A}_{j}{\varphi }_{{ij}}\right)}^{2}$$

The method was tested with ^18^F and ^223^Ra (Supplementary Fig. S3a, b).

### Microtome tissue preparation

For the two studies documented in this manuscript, mice were exsanguinated via cardiac puncture after being euthanized via CO_2_ prior to tissue sample collection. After exsanguination, target tissues were harvested and snap-frozen on dry ice. Frozen tissue samples were then imbedded in mounting media using Tissue-Tek O.C.T Compound (Sakura Fineteck USA, Torrance, CA, USA) and placed in the Leica CM3050 S Cryostat chamber (Leica Biosystems, Deer Park, IL, USA) to retain temperature (−20 °C) prior to sectioning. Sections between 10 and 50 microns (depending on study specifications) were collected on New Silane Adhesive coated glass slides (Newcomer Supply, Middleton, WI, USA) and placed into plastic slide boxes to dry prior to imaging. Duplicate “sister-sections” were collected for all tissue slices to ensure accuracy in comparison between all imaging methods. All mouse procedures were conducted in accordance with animal protocol approved by the National Cancer Institute Animal Care and Use Committee.

### T cell study

Naïve T cells were purified from C57BL/6J mice (Jackson Laboratory, Farmington, CT, USA) using CD8 and CD4 magnetic beads according to the manufacturer’s instructions (Miltenyi Biotec, Bergisch Gladback, Germany). ^89^Zr-oxine was generated in-house as previously reported^18,19^ and used for labeling the purified T cells by mixing the T cell suspension in PBS with ^89^Zr-oxine solution as described previously^18^. 9.47 × 106 labeled T cells, with a specific activity of 17.6 kBq/million cells, were intravenously injected into the mice. One day later, mice were euthanized, exsanguinated, and 10 and 30 µm-thick frozen splenic sections were prepared. These tissue sections were imaged on the QPID and brightfield imaged at a X40 magnification using an Ocus 40 (Grundium, Tampere, Finland) microscope.

The QPID and brightfield images (Fig. 5a, b, e, f) were registered using the GIMP opensource image processing software package^20^. The brightfield image was opened first. The QPID image was opened as a separate layer in gray scale. The QPID image was then colorized, and contrast adjusted to enhance the high-density areas. A threshold was then applied to convert the pixels below the threshold to full transparency. The QPID image was then scaled, translated and rotated by eye to register the two images. Alpha blending was done to adjust how well the brightfield and QPID images displayed together.

ImageJ^21^ was used to draw the regions of interest (ROI) to measure the background, low- and high-density regions. The low- and high-density regions were selected at random throughout the image. Mann-Whitney test was used for the statistical analysis determining any significance between the low- and high-density samples.

### ^223^RaCl_2_ and Na^18^F bioequivalence study

The ^223^RaCl_2_ and Na^18^F bio-equivalence study was performed by injecting a set of female athymic nu/nu mice (Charles River Laboratories, Wilmington, MA, USA) with a dose mixture of 7.4 MBq (200 µCi) of Na^18^F and 0.11 MBq (3 µCi) of ^223^RaCl_2_. The Na^18^F was purchased from Cardinal Health (Doublin, OH) prepared in their Beltsville MD facility. The ^223^RaCl_2_ was prepared from a ^227^Th sample purchased from Oak Ridge National Lab (Oak Ridge, TN) and purified in-house using a column prepared from an AG MP-1 resin (Bio-Rad Laboratories, Inc., Hercules, CA) to separate the ^223^Ra from the ^227^Th. The ^227^Th residual contamination was undetectable in the ^223^Ra sample using a high purity Ge (HPGe) detector measuring the ^227^Th 236 keV and 256 keV emission peaks over a period of 340 s. The column was packed from a slurry of the resin in water and then equilibrated with 8 M nitric acid. A solution of the ^227^Th in 8 M nitric acid was loaded unto the column and then eluted with 8 M nitric acid. The ^223^Ra solution which was contained in the void volume was evaporated to dryness. The dried residue was dissolved in saline and formulated with the Na^18^F for injection into the mice. The mice were sacrificed one hour post injection after which femur, spine and skull samples were prepared at 10, 20, 30 and 50 micron thicknesses. Only the spine samples were used for the bio-equivalence study.

To measure the injected activity, a 10 µL sample of the ^223^RaCl_2_ and Na^18^F mixture was aliquoted from the mixed solution sample and energy spectra and count rates were recorded by the microdose calibrator. The dual isotope deconvolution method was used to obtain concentration ratios of the two isotopes from which the total injected activity was measured. The residual was measured by placing the syringe post injection into the microdose sampling well and recording the energy spectra and count rate. The injected activity was corrected for the residual for this analysis.

Three mice were used for this bio-equivalence study. For each mouse, 6 separate spine sections were made at 10, 20, 30 and 50 µm thicknesses for a total of 24 microtome samples per mouse. Because of the short half-life of ^18^F and since only one microtome sample could be imaged at once with the QPID, the study was paced by injecting one mouse per day.

Once the 24 tissue samples were prepared, each sample was placed in the microdose calibrator for 2–5 min to collect and store the energy spectrum. The sample was then imaged with the QPID for 5 min, then imaged with the bright field microscope Ocus 40. The result is each tissue sample was measured in both the microdose calibrator and the QPID a few minutes apart.

Separate alpha and positron PID images were reconstructed from the QPID data resulting in an activity measurement for ^223^RaCl_2_ and Na^18^F content in the tissue sample. Using the corresponding microdose calibrator energy spectra, the energy spectra isotope deconvolution analysis was performed resulting in independent activity measurement of the two radio-compounds. All activity measurements were decay-corrected to the injection time. The percent injected activity was then calculated by dividing the decay-corrected ^223^RaCl_2_ and Na^18^F activity measurements from the QPID and microdose calibrator with their corresponding injected activity.

The bioequivalence referred to as B between ^223^RaCl_2_ and Na^18^F is defined for this work as

3 $$B=\frac{{\scriptstyle{223}\atop} \! {\mbox{RaCl}}_2\, \% {\mbox{IA}}}{{\mbox{Na}}^{18}{\mbox{F}}\, \% {\mbox{IA}}}$$

When measuring the ratio of ^223^RaCl_2_ or Na^18^F activity measured by the QPID and microdose calibrator, the calculated %IA for each nuclide and each tissue is used to calculate the ratio. The IA component cancels out.

4 $$R=\frac{{\mbox{QPID}} \% {\mbox{IA}}}{{\mbox{Microdose}} \% {\mbox{IA}}}=\frac{{\mbox{QPID Actvity}}}{{\mbox{Microdose Activity}}}$$

Graphs of QPID and microdose calibrator bioequivalence as a function of sample thickness were generated using Eq. (3) (Fig. 6g). The activity ratio comparing the β^+^ and α event rates between the QPID and microdose calibrator as a function of sample thickness were generated using Eq. (4) (Fig. 6h).

### Reporting summary

Further information on research design is available in the Nature Portfolio Reporting Summary linked to this article.

## Supplementary information

Supplementary Information

Reporting Summary

## Data Availability

Data sets generated during the current study are available from the corresponding author on reasonable request. The image data sets are raw binary form and analyzed using ImageJ. The raw data acquired from the QPID is too complex to provide written documentation, but the authors will work with interested parties if they desire access to the raw data and the purpose of the use of the raw data is for further understanding of the work presented herein. Use of the raw data for private or commercial purposes will have to be cleared with the Technology Transfer Division of NCI through their licensing program.

## Code availability

Source code used to generate the data presented in this manuscript is available from the corresponding author on reasonable request. Sharing of the source code will have to be cleared with the NCI Technology Transfer Division and may involve CRADA agreements or other licensing options.
