# Supplementary material for: A Quantitative Particle Identification (QPID) spectral autoradiography system
Source: Commun Eng. 2025 May 15;4:89. doi: 10.1038/s44172-025-00426-1 (PMC12081844; doi:10.1038/s44172-025-00426-1)
Supplement: Supplementary file 1 — Supplementary Information [file 44172_2025_426_MOESM1_ESM.pdf]

A Quantitative Particle Identification (QPID) Spectral Autoradiography System

Supplemental Material

Stephen S Adler[1], Noriko Sato[2], Kwamena Baidoo[2], Frank I Lin[2], Woonghee Lee[2], Colleen P Olkowski[2], Freddy E Escorcia[2], Peter L Choyke[2]

[1]Clinical Research Directorate, Frederick National Laboratory for Cancer Research, Frederick Maryland.
[2]Molecular Imaging Branch, National Cancer Institute, Bethesda Maryland.

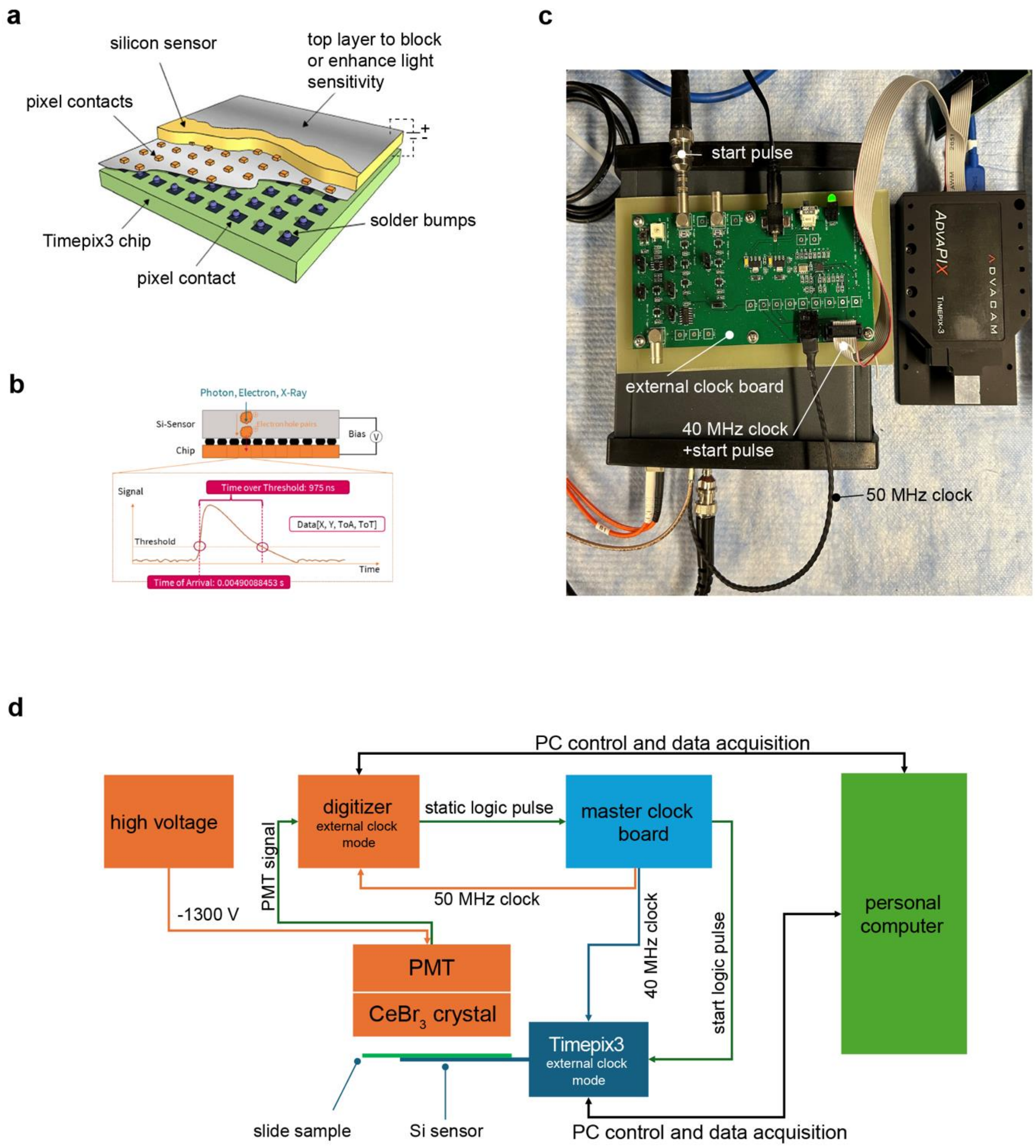

Figure S1. QPID architecture details. **a**, Cartoon of the Timepix3 sensor showing how the silicon wafer is bonded to the ASIC through the 55 um pitch pixel contacts. **b**, Diagram of the time over threshold measurement technique used to measure the ionization pixel time and magnitude of the ionization energy. **c**, Photo of the external clock circuit providing the 50 MHz and 40 MHz external clocks to the DT5725 digitizer and Timepix3 unit respectively. It also receives the start pulse from the DT5725 and sends it to the Timepix3 unit along with the 40 MHz clock over the ribbon cable. **d**, Schematic of the interconnected signals operating the QPID. Figures **a** and **b** were provided with permission from Amsterdam Scientific Instruments (Amsterdam, The Netherlands).

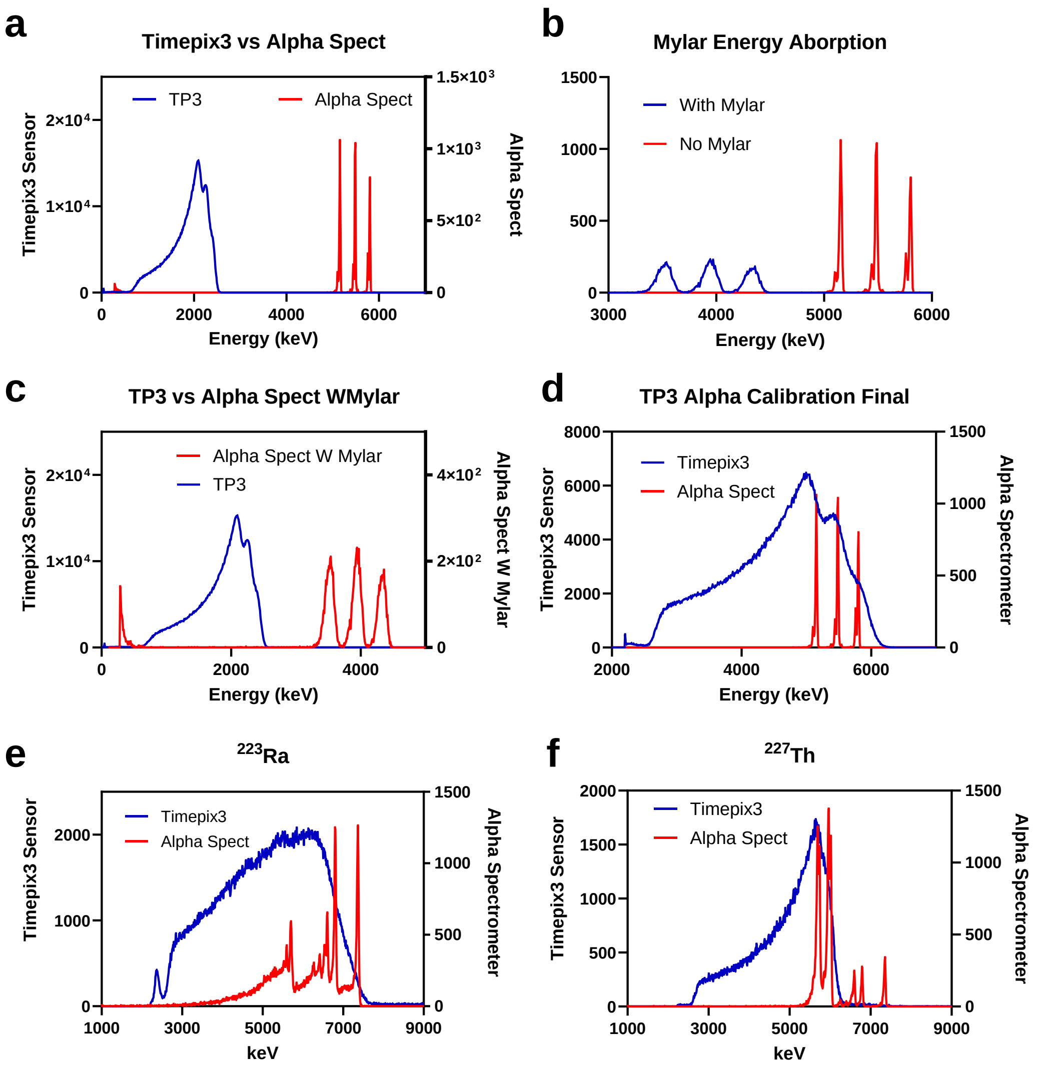

Figure S2. Alpha energy calibration results. a, Energy spectra plots showing the need for a dedicated α particle energy calibration. In blue is the energy spectrum of a ^{239}Pu, ^{241}Am and ^{244}Cm mixed source measured by the Timepix3 sensor with the factory energy calibrations applied. In red is the energy spectrum from the same mixed source measured with an α spectrometer. **b**, The effects of the mylar film used to protect the Timepix3 sensor from contamination. The mixed source was measured in the α spectrometer with and without the mylar film covering the source. The α particles lose about 1.5 MeV to 2 MeV energy when they traverse the mylar film. **c**, Timepix3 energy spectrum using the factory energy calibration and the α spectrometer. The mylar film is covering the source in both cases. This is the direct comparison of the Timepix3 ionization response of the α particles with an α spectrometer. **d**, The results of the secondary α calibration applied to the Timepix3 α particle tracks compared to the equivalent data measured by the α spectrometer. **e,f**, The Timepix3 Energy spectra after applying the full α calibration from a ^{223}Ra and ^{227}Th source respectively. The ^{227}Th source was measured several days after purification therefore one can see the start of the ingrowth of the ^{223}Ra and other daughters within the ^{227}Th decay chain.

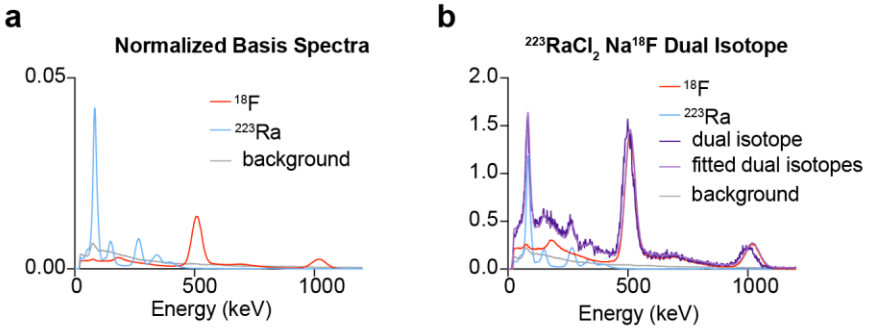

Figure S3. Microdose calibrator mixed source deconvolution example. **a**, Energy histogram basis functions used in the activity decomposition fit. **b**, The result of the energy histogram fit where the activity for each of the basis energy histograms has been determined and the sum of the three equals the measured dual isotope energy histogram. The ability to measure the activities of a mixed source using the microdose calibrator is key in doing quantitative analysis with the QPID. Because the activity measurements from the QPID and the microdose calibrator were not the same for ^{223}Ra but were the same for ^{18}F lead the study of bio-equivalence as a function of tissue thickness. The ^{223}Ra activity measured by the microdose calibrator was on average 79% ± 8.3% higher than what the measured by the QPID. This compares to 7.6% ± 2.8% average higher ^{18}F activity measured by the QPID compared to the microdose calibrator.

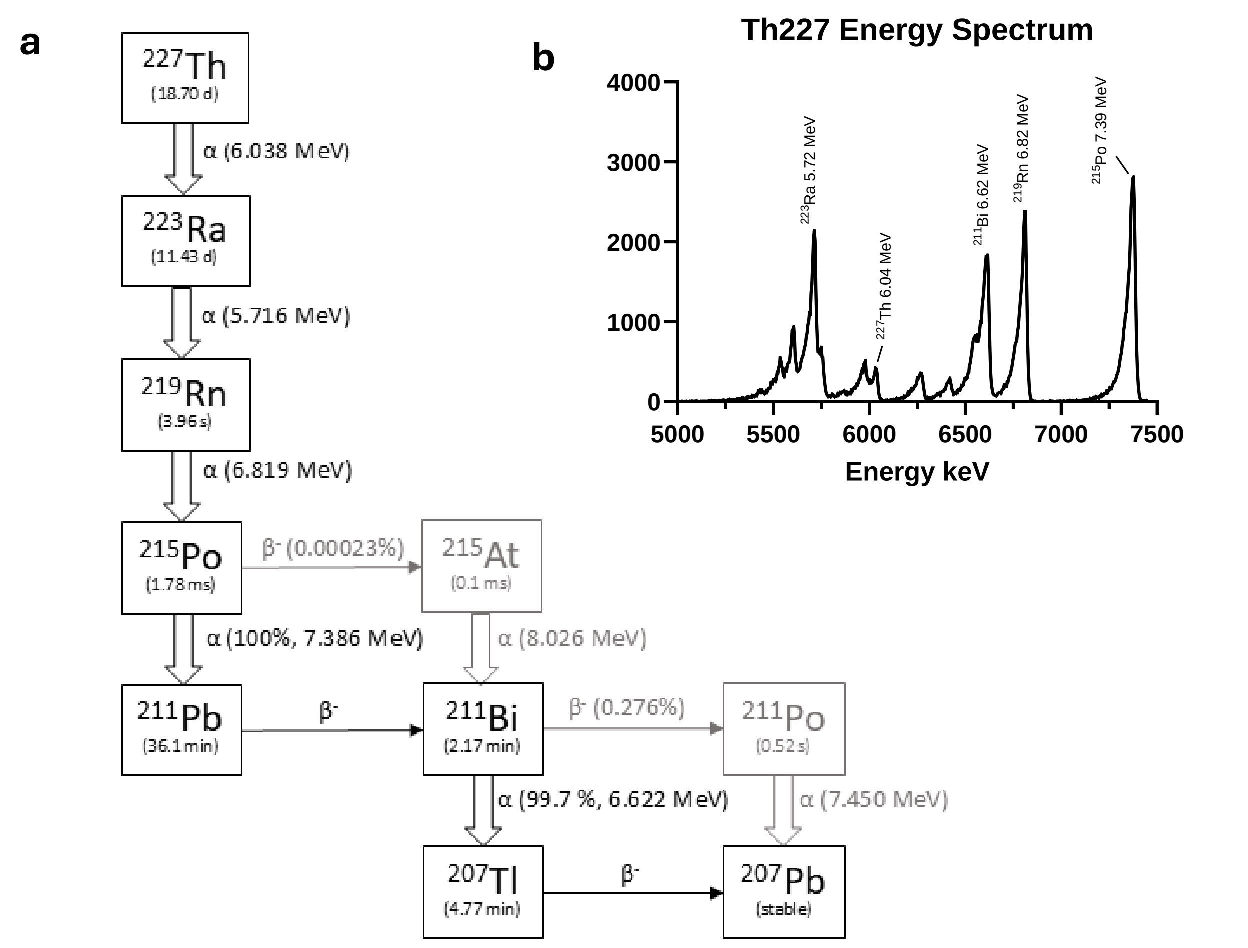

Figure S4. **a**, The ^{227}Th decay chain is presented. The energy of the highest energy and most predominant alpha emission is listed for the alpha decay radionuclides along with the half-life. In grey are the alternate decay paths which have a very small branching ratio and therefore are very hard to detect and to first order can be ignored. **b**, An α spectrometer energy spectrum of a ^{227}Th sample 2 months after purification thus showing the predominant alpha emission peaks of the 5 radioisotopes which form the ^{227}Th decay chain.

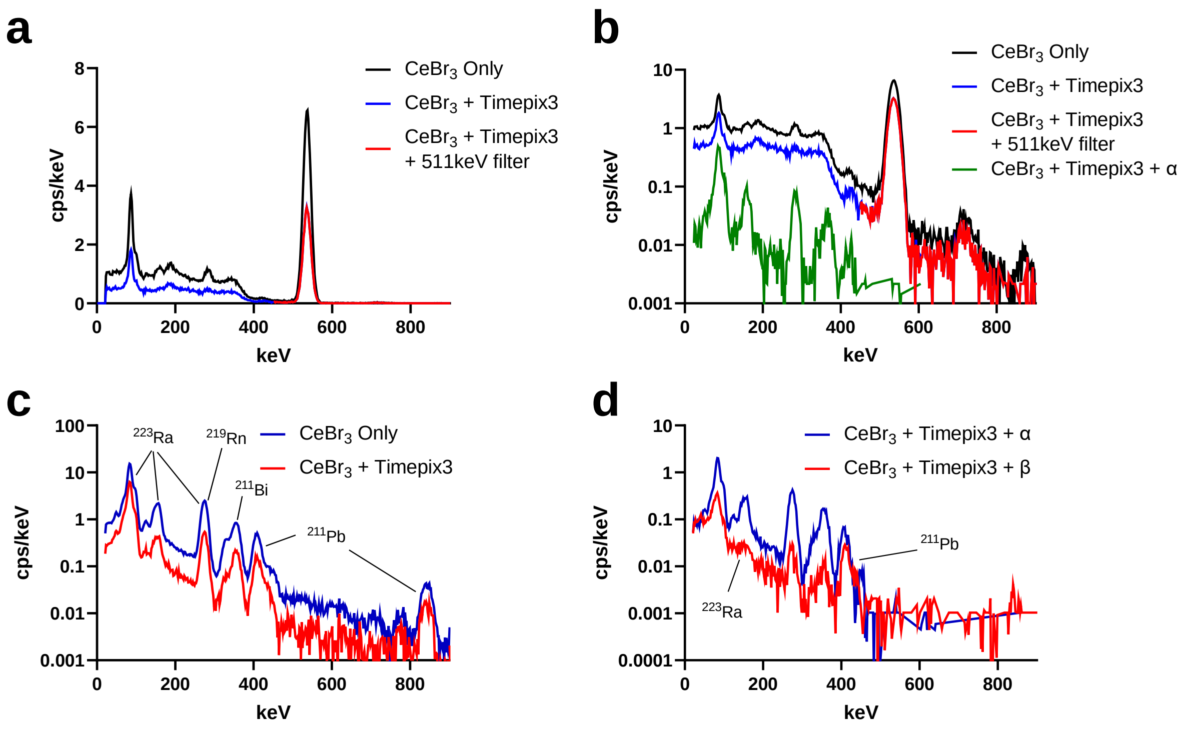

Figure S5. $CeBr_3$ energy spectra for various experimental conditions. **a**, Dual source sample of $^{223}RaCl_2$ and $Na^{18}F$. The spectrum drawn in black is generated from the raw $CeBr_3$ crystal data. The spectrum in blue and red is generated by requiring a coincidence with a particle track recorded by the Timepix3. No particle ID filters are applied. The red part of the spectrum is used to tag β^+. **b**, The same spectrum as in **a**, but displayed on a $\log_{10}$ scale. The spectrum drawn in green has an additional requirement that the Timepix3 particle tracks are made by α particles thus showing the gamma emissions made from the different daughter radionuclides forming the ^{223}Ra decay chain. **c**, The energy spectra displayed are from a pure $^{223}RaCl_2$ sample showing the raw data from the $CeBr_3$ in blue and with the additional Timepix3 particle track coincidence requirement in red. The various gamma emissions peaks are identified. **d**, Further processed spectra from the $^{223}RaCl_2$ sample with additional particle ID filters applied. The spectrum in blue shows the α tagged emissions while the spectrum in red shows the β^- tagged emissions. Notice the suppression of the ^{223}Ra peak in the red spectrum while the ^{211}Pb peak is not suppressed as much. The two spectra in **d** show the effect of selecting between α and β^- emissions using the Timepix3 and the resulting spectra measured by the $CeBr_3$ crystal. When selecting β^- tracks in the Timepixe3, one can see a suppression of the gamma peaks emitted through α decay, especially the 154 keV ^{223}Ra peak. There is still leakage in the ^{219}Rn and ^{211}Bi peaks. These are preliminary results with more work on this type of analysis will continue.
